# Recurrent colon cancer in a patient with Muir–Torre syndrome: a case report

**DOI:** 10.1093/jscr/rjae015

**Published:** 2024-02-06

**Authors:** Angeline C Rivkin, Philip Bystrom, Amy Y Lin, Vivek Chaudhry

**Affiliations:** University of Illinois at Chicago College of Medicine, 1853 W Polk St, Chicago, IL 60612, United States; University of Illinois at Chicago College of Medicine, 1853 W Polk St, Chicago, IL 60612, United States; University of Illinois Metropolitan Group Hospitals, Department of Surgery, 836 W Wellington Ave Room 4807, Chicago, IL 60657, United States; University of Illinois Hospital and Health Sciences System, Department of Pathology, 1740 W Taylor St, Chicago IL 60612, United States; University of Illinois Hospital and Health Sciences System, Department of Colon and Rectal Cancer Surgery, 1740 W Taylor St, Chicago, IL 60612, United States

**Keywords:** Muir–Torre syndrome, Lynch syndrome, hereditary nonpolyposis colorectal cancer syndrome, sebaceous carcinoma, skin cancer, colon cancer

## Abstract

Muir–Torre syndrome (MTS) is a rare subtype of hereditary nonpolyposis colorectal cancer syndrome caused by a defect in DNA mismatch repair leading to microsatellite instability. It is characterized by the presence of at least one sebaceous gland tumor and one internal malignancy, most commonly colorectal and endometrial tumors. These patients have a high propensity for tumorigenesis, and while strict screening protocols are in place, there are only two cases that describe the management approach to recurrent colon cancer. Here, we present a case of recurrent colorectal cancer in a patient with MTS, and describe how it was managed at our facility by a multidisciplinary team.

## Introduction

Muir–Torre syndrome (MTS) is a hereditary cancer syndrome caused by a defect in DNA mismatch repair (MMR) and is considered a variant of Lynch syndrome aka Hereditary Nonpolyposis Colorectal Cancer Syndrome (HNPCC). About 70% of MTS patients have MMR gene mutations (90% MSH2, 10% MLH1), and 30% have MUTYH mutations [1]. These mutations inhibit the repair of DNA base-pair mismatches, which accumulate in the non-coding regions of chromosomes and result in microsatellite instability [2].

Patients with HNPCC develop various internal malignancies, most commonly colorectal and urogenital tumors (specifically endometrial cancer) [2, 3]. MTS is distinguished by the presence of at least one sebaceous gland tumor (adenoma, carcinoma, keratoacanthoma, or basal-cell carcinoma) in addition to one or more internal malignancies. In contrast to sporadic colon cancer, Muir–Torre associated lesions develop 15–20 years earlier and tend to be more proximal [2].

The treatment of colorectal tumors in the context of MTS follows the standard multi-disciplinary approach to any colorectal cancer, resection being first-line with use of adjuvant chemotherapy and radiation when appropriate [4]. However, the approach to recurrent colorectal tumors in patients with MTS is poorly described in the literature. We present a case report detailing our approach to a patient with MTS with recurrent colorectal cancer after prior resection.

## Case report

Our patient is a 58-year-old Hispanic male with a history of hypertension, pulmonary embolism, iron deficiency anemia, and a history of colon adenocarcinoma at age 40 at the hepatic and splenic flexures, status-post right hemicolectomy with primary anastomosis. He has no known family history of cancer on either side and did not undergo genetic testing at the time of his initial cancer management.

**Figure 1:**
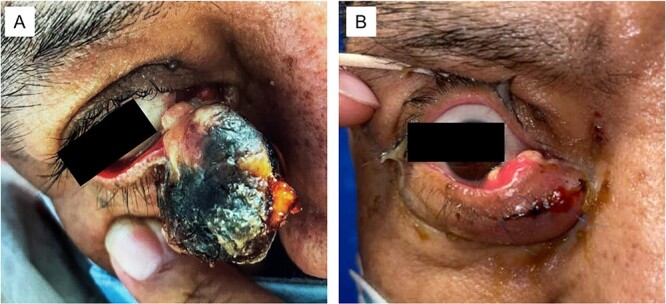
(A) Large sebaceous carcinoma of the right lower medial eyelid measuring ~2 × 3 cm. The fungating lesions appears necrotic with a light tan nodular base. (B) Sebaceous carcinoma 6 months post-excision with recurrent tan nodular lesions on the conjunctival surface, which were later re-excised during a conjunctival map biopsy procedure.

**Figure 2:**
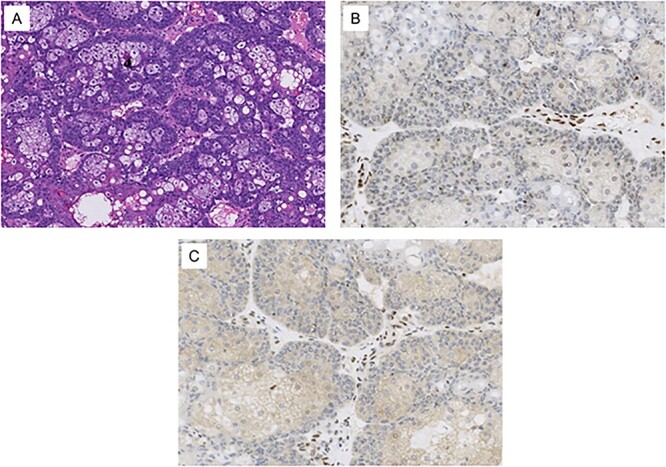
Histological images from the patient’s conjunctival map biopsy procedure. (A) H&E stained section showing tumor cells, some with vacuolated cytoplasm and others with a basaloid appearance. These features are consistent with well-differentiated sebaceous carcinoma. (B) Immunohistochemical staining for MLH1 shows loss of expression in the tumor cell nuclei. (C) Immunohistochemical staining for PMS2 shows loss of expression in the tumor cell nuclei. Normal stromal nuclei appear brown in images B and C, consistent with intact MLH1 and PMS2 staining.

He initially presented to oculoplastics for a mass of the right lower eyelid that had been enlarging over 3 months (Fig. 1); biopsy showed sebaceous cell carcinoma with genetic testing showing loss of MLH1 and PMH2 expression (Fig. 2). These findings, in conjunction with his prior colorectal cancer, resulted in his diagnosis with MTS.

A few months prior to his presentation, he had undergone routine radiographic surveillance with Positron Emission Tomography (PET)/CT and colonoscopy at an outside hospital, and was found to have both a left renal kidney mass and a 5 cm irregular, ulcerated, and friable mass near the site of his prior surgical anastomosis, which was obstructing the majority of the lumen. Biopsy of the colonic lesion was consistent with mucin-producing adenocarcinoma with signet ring features.

The patient was scheduled to undergo a complete colectomy with left nephrectomy but presented to the emergency department the night prior to his scheduled surgery for hematochezia. He was anemic with a Hb of 6.7 but otherwise hemodynamically stable. He was given one unit of packed red blood cells and taken to the operating room the following morning for exploration and resection of the tumors.

He was found to have a firm mass at the site of the ileocolic anastomosis with two adherent loops of small bowel. The mass was not tethered to any other structures. He underwent a total abdominal colectomy with primary anastomosis as well as resection of the involved loops of bowel with primary anastomosis. Urology performed a left radical nephrectomy which was complicated by significant bleeding from a renal vein injury which was ultimately controlled with the assistance of vascular surgery. Estimated blood loss was 2.5 L.

Following completion of the case the patient was transferred to the Intensive Care Unit on mechanical ventilation. He was extubated on postoperative Day 1 without issue. The remainder of his hospital stay was unremarkable, and he was discharged home on postoperative Day 4. Histologic studies of the colon cancer specimen revealed a heterozygous mutation in the MLH1 gene, consistent with MTS. Pathologic analysis of the renal mass was consistent with T2aNx clear cell carcinoma with negative resection margins.

## Discussion

MTS is a rare hereditary cancer syndrome that requires treatment by a multidisciplinary team to manage the various tumor types with multimodal therapies. In our case, the patient’s care team consisted of physicians from multiple specialties including colorectal surgery, urology, ophthalmology, dermatology, hematology, radiation oncology, and medical genetics.

A robust and stringent screening program plays a crucial role in the management of these patients given their propensity for tumorigenesis. Patients with MTS should follow the tumor screening guidelines that have been established for HNPCC. According to the American College of Gastroenterology’s clinical guidelines, patients should have a colonoscopy at least every 2 years starting at age 20–25, annual screening for endometrial and ovarian cancer by biopsy and transvaginal ultrasound when appropriate, upper endoscopy at baseline, and all regular recommended population-based cancer screening (e.g. for prostate, breast) [4]. Baseline and annual PET/CT scans may be considered, as nearly 50% of patients with MTS have two or more internal malignancies [5].

Though patients with MTS may present with multiple different internal and cutaneous malignancies throughout their lives, there are very few cases describing the management of recurrent colorectal cancer after previous resection in these patients. Two cases were found that describe recurrent intestinal cancer near the sites of previous resection; one at the anastomotic site and one 10 cm from the ileostomy [6, 7]. One additional interesting case describes a patient who underwent radical cystoprostatectomy with neobladder creation from the ileum for urothelial carcinoma, who later developed both invasive colon adenocarcinoma and recurrent urothelial carcinoma of the neobladder [8]. All three of these patients were treated with repeat resection, and were still alive at the time of their case publications 2–16 years later.

These cases, including the case presented here, demonstrate that recurrent colorectal cancer in the setting of MTS can be treated like recurrent cancer from any other cause. Each new tumor requires individual management based on its location and stage. Due to the high propensity for tumorigenesis in multiple locations, regular imaging surveillance and a multidisciplinary approach is vital for the management of patients with MTS.
